# Occurrence, Distribution, and Ecological Risk Assessment of Antibiotics in Selected Urban Lakes of Hanoi, Vietnam

**DOI:** 10.1155/2021/6631797

**Published:** 2021-03-10

**Authors:** Hong Anh Duong, Thi Vi Phung, Thuy Ngoc Nguyen, Lan-Anh Phan Thi, Hung Viet Pham

**Affiliations:** ^1^Research Centre for Environmental Technology and Sustainable Development, VNU University of Science, Vietnam National University, Hanoi 334 Nguyen Trai, Thanh Xuan, Hanoi 100000, Vietnam; ^2^Key Laboratory of Analytical Technology for Environmental Quality and Food Safety Control, VNU University of Science, Vietnam National University, Hanoi 334 Nguyen Trai, Thanh Xuan, Hanoi 100000, Vietnam

## Abstract

Residue concentrations of fifteen antibiotics including sulfonamides, quinolones, macrolides, *β*-lactams, and trimethoprim in lakes from Hanoi metropolitan area, Vietnam, were analyzed using ultra-performance liquid chromatography coupled with tandem mass spectrometry (UPLC/MS-MS) to elucidate their occurrence and behavior in urban environment. For surface water, the average concentrations of five antibiotic classes decreased in the order: sulfonamides (117.9 ng/L) > *β*-lactams (31.28 ng/L) > quinolones (20.19 ng/L) > macrolides (17.74 ng/L) > trimethoprim (8.93 ng/L). While the highest concentration of SMX was detected at 806.5 ng/L in surface water, those obtained in sediment were only at 1.35 ng/g because of their high solubility in water. Quinolones were found at a maximal concentration of 158.7 ng/L for OFL in water phase whereas those in sediment phase were 4,017 ng/g due to their great affinity in sediment. These findings revealed the different fate and release mechanisms of each antibiotic group in the environment. The ecological risk assessment implied some targeted compounds, and in particular, OFL and AZM could pose high risks to algae in the aquatic ecosystem.

## 1. Introduction

Antibiotics are widely used to protect the health of humans and animals or to promote growth rate of animals as food additives and antibiotic usage in the world exceed 200,000 tons per year and antibiotic consumption is on the rise [1]. Although antibiotics are extensively prohibited as growth promoters in farming and husbandry, they may be used in regular infectious diseases. It is undeniable enormous benefits of antibiotics in the treatment of bacterial infection for humans and animals. However, the development of the healthcare sector entails considerable consequences associated to antibiotics due to their toxicity to living organisms and the occurrence of antibiotic resistance genes (ARGs) and antibiotic resistant bacteria (ARB) in the aquatic environment. For this reason, antibiotics have been categorized as emerging contaminants which may contaminate and cause the adverse impacts to the environment, in particular freshwater ecosystems including water, sediment, and aquatic organisms. Between 50% and 90% of antibiotics used in humans and animals are excreted via urine and feces as a mixture of parent and metabolite form into the environment via waste streams or stormwater runoff [2, 3]. As a result, antibiotics have been detected in all of environmental compartments, e.g., in groundwater [4], drinking water [5–7], wastewater [7], surface water [8–10], sediment [11–13], and sludge [12]. It is inevitable that aquatic organisms have the potential to be exposed to ecotoxic effects of these unexpected residual antibiotics through the food web beginning with first consumer, e.g., algae [13], which means it may threaten to the global public health and the ecological security. The environmental risk of antibiotics to aquatic organisms has been well reported in some high-income countries, but it has been scarce in developing countries such as Vietnam.

Vietnam is the world's 15th most populous nation with a population of over 96 million in 2019. In the current context, the demand for antibiotics has increased rapidly as a consequence of high economic growth, urbanization rate, and higher urban population [8]. In fact, there was a double increase between 2009 and 2015 in antibiotic consumption with very high rate of resistance antibiotics in Vietnam [14]. It is remarkable that antibiotics and other pharmaceuticals in Vietnam can be easily purchased at any pharmacy without prescription and inappropriate use of antibiotic in developing countries was reported in the previous literature [15]. Meanwhile, the widespread occurrence of antibiotics was detected in the environment, e.g., surface water [8], wastewater from hospital [16, 17], aquaculture [18, 19], husbandry [20, 21], and pharmaceutical manufacturer [20]. However, data are limited about their fate after release in the stream and behavior through different environmental conditions. Therefore, significant attention is being paid to assess their effects and ecological risks of antibiotics in the environment in Vietnam.

West Lake and Yen So Lake are two largest urban reservoirs in the middle of Hanoi capital, Vietnam. For many years, these catchments have gradually been important water sources for aquaculture activities to serve the need of local residents. While the Vietnam government has struggled with alarming environmental pollution issues in Hanoi City with the second highest population density in Vietnam, West Lake and Yen So Lake still play a significant role as detention basins which daily receive a large amount of municipal treated/untreated wastewater from urban canals. Until now, there have been a few reports monitoring antibiotic residues in surface water and their ecological risks in Hanoi, and even no reports on their fate and transportation in sediment phase. In recognition of these concerns, this study aims to provide a comprehensive analytical discussion regarding the presence, distribution, and fate of antibiotic residues in environmental samples including surface water and sediment samples in West Lake and Yen So Lake in Vietnam. Additionally, the ecological risk of antibiotics was assessed through risk quotients in terms of filling the existing knowledge gap.

## 2. Materials and Methods

### 2.1. Standards and Chemicals

The target compounds belong to 5 families: (1) sulfonamides (SAs), including sulfamethoxazole (SMX), (2) quinolones (QNs), including ofloxacin (OFL), ciprofloxacin (CIP), moxifloxacin (MXF), and norfloxacin (NOR), (3) macrolides (MLs), including clarithromycin (CLR) and azithromycin (AZM), (4) *β*-lactams (*β*-Ls), including cefixime (CFM), cefotaxime (CTX), cefaclor (CEC), cephalexin (CFX), cefadroxil (CDX), amoxicillin (AMX), and ampicillin (AMP), and (5) trimethoprim (TMs), including trimethoprim (TMP). Fifteen target antibiotics were purchased from Sigma-Aldrich (the USA) and National Institute of Drug Quality Control (Vietnam). Furthermore, ofloxacin-d3, azithromycin-d3, and cefotaxime-d3 sodium are surrogate standards for the selected antibiotics, and sulfamethoxazol-d4 was chosen as an internal standard for the quantification of all the samples.

### 2.2. Study Area and Sampling Collection

Two sampling sites were conducted in Hanoi capital including West Lake and Yen So Lake in 2019. West Lake is the largest lake in Hanoi with the shore length of 17 km and an area of 530 ha. Unlike developed countries, wastewater collection and treatment systems in low-income and middle-income countries are still inadequate. Being a significant location for recreation with a lot of surrounding gardens, pagodas, hotels, villas, and other amusement parks, West Lake reluctantly becomes an unwanted reservoir with over 30 aqueducts receiving wastewater directly from neighborhood. Likewise, Yen So Lake is a complex of small and big reservoirs lying in the South of Hanoi with a water area of 70 ha. A considerable amount of municipal wastewater from the rivers such as To Lich, Kim Nguu, and Set flows into Yen So Lake. In fact, a part of wastewater from these rivers is treated at Yen So wastewater treatment plant located beside the lake with a capacity of 200,000 m^3^/day before flowing directly into Yen So Lake. It is worth mentioning that aquaculture activities in two lakes regularly provide fish for local markets.

**Figure 1:**
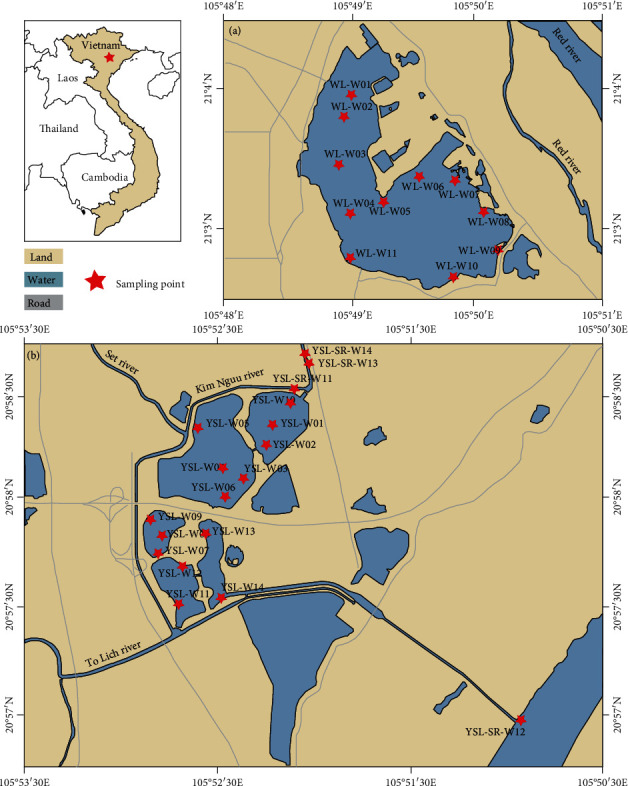
Sampling sites for urban lakes. Sampling points WL-W01 to WL-W11 are located in West Lake (a). YSL-W01 to YSL-W14 are situated in Yen So Lake (b).

The sampling sites are shown in Figure 1. Briefly, water and sediment samples were taken at each point, WL (*n* = 11), YS (*n* = 14). The samples were kept in clean containers at 4°C before treatment and analysis.

### 2.3. Analysis of Antibiotics

Concentrations of sulfonamide, quinolones, macrolides, *β*-lactams, and trimethoprim were determined by tandem mass spectrometry equipped with high-performance liquid chromatography (LC-MS/MS) according to the method 1696 entitled pharmaceuticals and personal care products in water, soil, sediment, and biosolids by HPLC/MS/MS [21]. Each 500 mL of water sample was filtered using a 0.45 *μ*m glass fiber membrane and acidified to pH 2 before extraction. Next, 50 *μ*L of the mixed surrogate solution (ofloxacin-d3: 2 ppm, azithromycin-d3: 2 ppm, cefotaxime-d3 sodium: 20 ppm, and sulfamethoxazol-d4: 5 ppm) and Na_2_EDTA (500 mg) was added to the water sample. An Oasis HLB cartridge (6 mL, 500 mg, Waters, the USA) was conditioned with 10 mL of methanol, followed by 6 mL of deionized water and 6 mL of HCl (pH 2) before the water sample was added and loaded through a HLB cartridge with a flow rate at 5–10 mL/min. Then, the cartridge was washed by 10 mL of deionized water and vacuum-dried for 3–5 min. The following step was that the targeted compounds were eluted with 6 mL of methanol and 2 mL of a mixture of acetonitrile and methanol (1 : 1 v/v). Finally, the extract was concentrated to 1 mL under nitrogen stream and analyzed by LC-MS/MS.

For the sediment sample, approximately 5.0 g of wet sample was spiked with 50 *μ*L of the surrogate standard, 15 mL of phosphate buffer (0.2 M, pH 2), and 20 mL of acetonitrile. The sample then was sonicated for 5 min, shaken with a horizontal shaker at room temperature for 30 min, and centrifuged at 1500 rpm for 15 min. This extract process was repeated in twice. The whole extracts were combined and evaporated to 20 mL by a vacuum rotary evaporator. Next, a solution mixture of Na_2_EDTA (500 mg) and deionized water (200 mL) was added to the extract. The solid phase extraction procedure was conducted to extract target antibiotics from sediment samples using HLB cartridges, and the following steps were the same as for water sample.

### 2.4. Liquid Chromatography-Tandem Mass Spectrometry Analysis

The analysis was carried out by UPLC-ESI-MS/MS 8040 instrument (Shimadzu, Japan) in positive ionization mode. The chromatographic separation was performed on a Poroshel-C18 column (15 cm × 2.1 mm i. d *x* 2.7 *μ*m, Agilent). The mobile phase included solvent A (0.3% formic acid (v/v) và 0.1% ammonium formate (m/v) in HPLC water) and the solvent B (acetonitrile: methanol (1 : 1 v/v)). The flow rate was hold at 0.25 mL/min with the following gradient program: the mobile phase was initially 95% A and 5% B in a period of 4 min and held for 18 min. Then, the rate of mobile phase A was decreased to 12% within 1 min and continued to reach 0% at min 23. This flow was kept for 3 min and increased to 95% of mobile phase A. The column and sample tray temperature were stable at 40°C and 4°C, respectively. Source and desolvation temperature were held at 140°C and 350°C. Desolvation and cone gas rate were 400 and 80 L h^−1^. The optimized parameters for each antibiotic are shown in Table 1.

### 2.5. Quality Assurance and Quality Control

To monitor procedural performance and matrix effects, the surrogate compounds were spiked with each sample. A procedural blank and a matrix spike sample were analyzed simultaneously with every 10 real samples to check for contamination of the analytical procedure. The instrument detection limit (IDL) and instrument quantitation limits (IQL) are described in Table 2. The limits of detection (LOD) and limits of quantitation (LOQ) were the concentrations which the ratio of signal-to-noise (S/N) was 3 and 10, respectively. In this study, LOD were in range of 0.12–0.75 ng/L for water sample and 0.05–0.15 ng/g dry weight for sediment. The recoveries for the antibiotics ranged from 62 to 115% in surface samples and from 65 to 126% in sediment samples.

### 2.6. Ecological Risk Assessment of Antibiotics

According to the European Chemicals Agency Guideline [22], the ecological risk quotient (RQ) was evaluated based on the ratio of the maximal measured environmental concentration (MEC) to the predicted no-effect concentration (PNEC) as depicted in equation (1). The lowest PNEC was used for the same species:

(1) $$\begin{array}{c} \text{RQ}=\frac{\text{MEC}}{\text{PNEC}}. \end{array}$$

There are three levels of the risk, low risk with the RQ ranging from 0.01 to 0.1, moderate risk with the RQ ranging from 0.1 to 1, and high risk with the RQ > 1 [23].

In addition, the PNEC value was determined by assessment factor (AF) using

(2) $$\begin{array}{c} \text{PNEC}=\frac{\left(\text{NOEC or EC}50\right)}{\text{AF}}, \end{array}$$

where NOEC is no observed effect concentration and EC50 is half maximal effective concentration.

## 3. Results and Discussion

### 3.1. Occurrence of Antibiotics in Surface Water

Among fifteen target antibiotics, TMP, SMX, OFL, and CLR were the most frequently detected compounds in 100% of surface water samples while MXF and CDX were hardly detected in water samples from WL. Detailed information about concentrations of antibiotics in surface water from West Lake and Yen So Lake is shown in Tables S1 and S2 in the Supplementary Information. Taken together, the average concentrations of five antibiotic classes decreased in the order: sulfonamides (117.9 ng/L) > *β*-lactams (31.28 ng/L) > quinolones (20.19 ng/L) > macrolides (17.74 ng/L) > trimethoprim (8.93 ng/L). This pollution trend can be explained that sulfonamides and *β*-lactams were used both for humans and animals while quinolones and macrolides are mainly used for humans [1]. The detection frequency of antibiotics in two lakes was 72% for AMP, 54% for CIP and MXF, 46% for CTX, and 29% for NOR and AZM. The total concentrations of 15 antibiotics ranged from 40.3 to 674.0 ng/L (mean: 253.9 ng/L) in West Lake and 101 to 1,753 ng/L (mean: 542.6 ng/L) in Yen So Lake. In general, most of the antibiotics were found in West Lake (WL) at lower concentrations compared to those in Yen So Lake (YS) which receives treated and untreated wastewater from urban rivers including Set River, Kim Nguu River, and To Lich River. In particular, these rivers which flow through urban areas play an important role to directly collect wastewater from households and commercial buildings in Hanoi. For individuals, statistically significant differences for SMZ, OFL, CLR, and TMP were observed between WL and YS (*p* < 0.05). The average concentrations of SMZ, OFL, CLR, and TMP were 7.51 ng/L, 33.2 ng/L, 1.75 ng/L, and 1.11 ng/L in WL whereas those in YS recorded at concentrations of 178.9 ng/L, 73.62 ng/L, 45.76 ng/L, and 13.27 ng/L, respectively. Depending on different pollution levels as well as characteristics of contamination sources, there is a variation in the concentrations of antibiotics between lakes [24].

A summary of antibiotics in surface water from two lakes and other studies around the world is shown in Table 3. The occurrence pattern of all investigated antibiotics in WL was similar to those in YS. In this study, sulfonamide which was one of the most abundant antibiotic classes was omnipresent in urban lakes with concentration ranging from 4.54 to 806.5 ng/L (mean: 117.7 ng/L). Likewise, it is reported that sulfonamide antibiotics were frequently detected in surface water in Vietnam [8], France [26], Spain [25], Germany [28], and China [27]. The high detection frequency of SMX could be the direct discharge from municipal activities in Hanoi, which reflects the wide usage of this antibiotic in densely populated cities. Besides, SAs are also known as compounds possessing the high solubility as well as good stability in water [27]. It is noteworthy that exceptionally high levels of SMX were found at site YS. W14-SR (806.5 ng/L), YS. W13-SR (459.9 ng/L), YS. W11-SR (269.9 ng/L), and YS. W12-SR (158.8 ng/L). In comparison to other sites in this study, all sites with notable high concentrations of SMX are located on the urban canals such as To Lich and Kim Nguu considered as the most polluted rivers in Hanoi. Moreover, two remarkable sites, YS. W14-SR and YS. W13-SR, are near the Yen So wastewater treatment plant (WWTP) where treated wastewater flows into Kim Nguu River afterwards. This observation for two greatest levels could be explained due to incomplete removal and the biotransformation of its acetylated form in anaerobic treatment unit [44]. In fact, certain literature documents illustrated that TMP is regularly used in combination with SMX for the purpose of improving the therapeutic effect [45]. That might be the reason why TMP and SMZ were found with relatively abundance pattern in this study and other studies [32]. The concentration of TMP ranged from 0.65 to 67.9 ng/L (mean: 8.93 ng/L) with a detection frequency of 100%, suggesting their widespread application for human treatment.

Despite the various regulations and management of antibiotics between countries, there were many similarities in the occurrence of antibiotics in surface water worldwide. In Vietnam, quinolones are extensively used for a variety of infections and this group is represented by OFL. For quinolones, OFL was the most dominant compound at a concentration range of 28.18 to 158.7 ng/L (mean: 59.19 ng/L) while CIP (mean: 1.33 ng/L), MXF (7.43 ng/L), and NOR (0.11 ng/L) were detected at lower concentrations. These findings were consistent with those reported in the previous studies in Vietnam [8], Latin America [29], and Brazil [6]. Of quinolone antibiotics, OFL was observed at very high concentrations in urban hospital wastewater [16], which means that OFL was widely used for human treatment. On the other hand, it is quite easy to purchase any medicine, even antibiotics, and self-medication is a common practice in Vietnam. Consequently, it suggests that households are a significant source of OFL to the waste stream. The lower pollution levels of these antibiotics can be due to their sensitivity to light and the adsorption towards sediments [1]. Besides, the use of quinolones such as CIP and NOR have been banned in aquaculture in Vietnam for a long time.

Concentrations of macrolides (CLR and AZM) in WL were in range of <MQL to 243 ng/L. Obviously, CLR appeared in all samples with an average concentration of 30.04 ng/L and the maximum level of CLR was 243 ng/L at site YS. W14-SR. By contrast, the low detection frequency of AZM (DF = 29%) can be attributed to the lower consumption of this antibiotic in urban areas. Due to the fact that the price of CLR (0.43 USD/tablet/250 mg) was significantly cheaper than AZM (5.19 USD/bottle/200 mg/5 ml) and CLR is one of the most common antibiotics in Vietnam [46], it is not surprising that concentrations of AZM were much lower than those of CLR in this study (*p* < 0.05). As a result, this presence pattern of AZM seems to be different from findings in developed countries, e.g., Singapore [37], Iran [38], and Spain [39], in which AZM was an abundant macrolide antibiotic with very high detection frequency.

Among seven investigated *β*-lactams, AMP was the most prevalent *β*-lactams (DF = 72%), followed by CTX (DF = 46%), AMX (DF = 25%), CFX (DF = 21%), and CEC (DF = 21%) with concentration ranges summarized in Table 1. The highest concentration of individual antibiotics was found at 1,572 ng/L for CFM in Yen So Lake (site YSL. W08). This study observed high levels of contamination by CEC and AMX at two sites, WL. W03 (CEC: 301.3 ng/L; AMX: 221.3 ng/L) and WL. W06 (CEC: 215.4 ng/L; AMX: 157.8 ng/L) in the West Lake. Being one of the most commonly sold antibiotics in Vietnam, AMP found at contamination levels ranging from undetected to 70.14 ng/L (mean: 32.27 ng/L) in two lakes potentially derives from consumption of human medicine and incomplete elimination during wastewater treatment. On the other hand, abundance of AMP can be explainable that AMP is one kind of over-the-counter medicines in Vietnam widely used for upper respiratory tract and gastrointestinal diseases due to its affordable price. For example, a statistical report in rural areas of Vietnam with low density population indicated that 62% of pediatric patients under five sought care at health facilities and drugstores for respiratory infections while *β*-lactams were used in 90% of cases [47]. In addition, nevertheless, CFM and CDX were rarely observed (DF = 7%) in surface water samples because of possibly rapid degradation in human body. The instability of *β*-lactams through adsorption, thermal degradation under sunlight, and enzymatic biodegradation has previously been demonstrated in the literature [48, 49]. Eventually, *β*-lactams readily degraded through hydroxylation, cleavage, and mineralization to carbon dioxide and water. Hence, *β*-lactams are not generally considered as serious environmental contaminants [49].

In general, *β*-lactam antibiotics were negligible or found at low concentrations which were similar to those reported in Australia [49], China [40], Egypt [41], and Ghana [42] in spite of their widespread usage pattern.

### 3.2. Occurrence and Distribution of Antibiotics in Sediment in Two Urban Lakes

**Figure 2:**
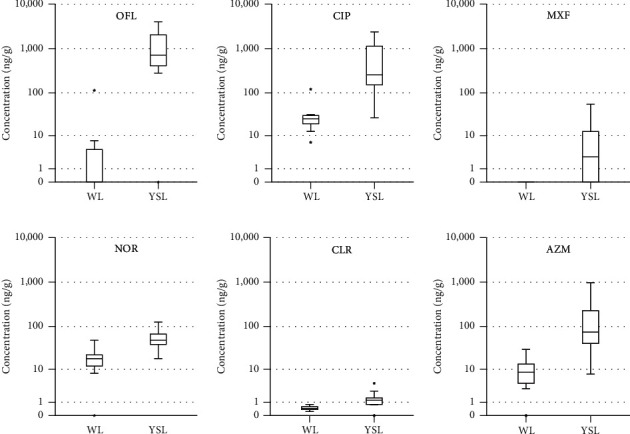
Box plot of antibiotic concentrations in sediment taken in West Lake (*n* = 11) and Yen So Lake (*n* = 14).

In sediment, only six antibiotics, OFL, CIP, MXF, NOR, CLR, and AZM, belonging quinolones and macrolides showed high detection frequencies while seven *β*-lactam antibiotics (CFM, CFX, CEC, CFX, CDX, AMX, and AMP) were absent and sulfonamides (SMX) and trimethoprim (TMP) were rarely found with low concentrations. Concentrations of fifteen antibiotics in sediment taken from West Lake and Yen So Lake were indicated in Tables S2 and S3 (Supplementary Information). Although there was a difference in concentrations of targeted compounds, depending on characteristics of waste sources and position of each site, the fate pattern of antibiotics in two lakes was persistent with those reported in around the world. The statistical two-sample *t*-test illustrated that significantly higher levels for OFL, CIP, MXF, NOR, CLR, and AZM were presented in Yen So Lake in comparison with West Lake (*p* < 0.05) (Figure 2).

Among five investigated groups of antibiotics, quinolones were ubiquitously detected in sediment with highest frequencies ranging from 64% to 100%, except for MXF which only was found in Yen So Lake. It was noted that the sorption of quinolones was remarkably strong compared to other antibiotics [50]. Concentrations of OFL varied from 3.51 ng/g to 113.9 ng/g (mean: 32.6 ng/g, DF = 36%, DF = 36%) in West Lake and ranged from 281.36 ng/g to 4,017 ng/g (mean: 1,416 ng/g, DF = 93%) in Yen So Lake. These observations in Yen So Lake were much higher than those detected in sediment in the Haihe River (mean: 36.8 ng/g) [1] and in the Fengshuba Reservoir (mean: 7.1 ng/g) in China [13] and Mississippi and Minnesota rivers in America [11]. Similarly, CIP was found in 100% of sediment samples with a median concentration of 31.91 ng/L and 668.5 ng/L in West Lake and Yen So Lake, respectively. NOR was the main detectable compound and its concentrations (level range: 57.32 ng/g to 124.9 ng/g; mean: 57.32 ng/g) in Yen So Lake were two orders of magnitude higher than those (level range: <MQL to 48.25 ng/g; mean: 18.58 ng/g) in West Lake. However, this research witnessed a great discrepancy in levels of MXF between two lakes. Indeed, the detection frequency of MXF was 64% of all sediment samples with a range of 0.14 ng/g to 24.68 ng/g whereas this compound was completely absent in West Lake. Generally, the pollution levels of quinolone antibiotics in sediment collected from West Lake were in consistence with other studies; meanwhile, those observed at Yen So Lake were found at significantly higher levels compared to the previous literature in other countries [1, 11, 12]. Unlike freshwater reservoirs in developed nations, Yen So Lake is the receiving body water for a large amount of major municipal wastewater in metropolitan areas of Hanoi. In addition, investigated sites with very high concentrations are far from drains where water circulation occurs in many bodies of water; hence, there was accumulation and deposition of antibiotics in sediment over time. It can be seen that quinolones and macrolides were detected at highest concentrations and detection frequencies in sediment. In addition to their wide consumption in human medicines, this can be explained by that quinolones with high the sediment-water distribution coefficient are easily adsorbed in sediment [10].

The occurrence of fluoroquinolones, sulfonamides, and tetracycline in sediment has been well documented in other countries. However, very little data on accumulation of macrolides such as AZM and CLR in sediment are available. For this reason, it is necessary to investigate and evaluate their occurrence pattern in sediment. In fact, the results of this study illustrated ubiquitous presence of selected macrolides in sediment, and especially, CLR and AZM were omnipresent with a DF ranging from 82 to 100%. Despite ubiquitous detection of macrolides, CLR in sediment showed the low concentrations varying from 0.23 to 0.79 ng/g (mean: 0.48 ng/g) in West Lake and from LOD to 4.33 ng/g (1.36 ng/g) in Yen So Lake. In general, the variation in the concentrations of CLR in two urban lakes seems to be similar to that reported in the Arc River in France [51]. Coincidentally, paired-sample *t*-tests indicated that there was a remarkable discrepancy in the concentrations of AZM between two urban lakes (*p* < 0.05) whereas AZM varied from 0.23 ng/g to 0.79 ng/g (mean: 0.48 ng/g) in sediment of West Lake and those were detected at much higher concentrations ranging from 7.56 ng/g to 969.2 ng/g (mean: 169.9 ng/g) in Yen So Lake. Concentrations of AZM in sediment collected from Yen So Lake were in the same order of magnitude as those found in France [51]. In addition, Feitosa-Felizzola and Chiron [51] reported that AZM was much more associated with sediment than CLR because of its greater hydrophobicity [51]. As a result, the accumulation trend of AZM in sediment was much higher than CLR. One of the factors contributing to high concentrations of antibiotics in sediment was the low flow condition [52]. In an earlier study, Kolpin et al. [53] proved that levels of different pharmaceuticals and organic wastewater contaminants including antibiotics varied with flow with the highest levels and DF found during low flow conditions (40%) in comparison with high (nearly 10%) and medium flow (nearly 10%) conditions [53]. Meanwhile, there is a great deal of flow through two urban lakes, especially Yen So Lake increasing the potential for pollutant accumulation not only in surface water but also in sediment.

Not surprisingly, *β*-lactams were not detected in any sediment samples of urban lakes in this study due to its absence in water phase, which is inconsistent with the results in China [13]. Likewise, SMX and TMP were rarely observed in sediment, which can be explained that SMX with lowest organic carbon-water partition coefficient (K_OC_) is the least hydrophobic compound [52]. That pollution trend in the antibiotic levels and composition of sediment showed different patterns compared with previous studies [1, 52, 54], in which detection frequencies of *β*-lactams, SMX and TMP, were significantly lower than those observed in China [10].

**Figure 3:**
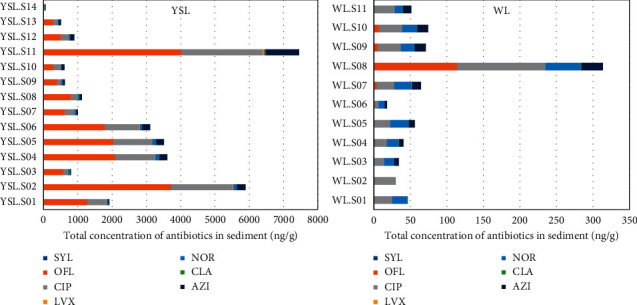
Distribution of antibiotics in sediment.

As shown in Figure 3, distribution of antibiotics in sediment taken from two lakes was evaluated, except for targeted compounds with negligible concentrations or no detection including CFM, CTX, CEC, CFX, CDX, AMX, and AMP. Generally, profile pattern of antibiotics in West Lake was slightly different than Yen So Lake, depending on a number of factors. The most predominant antibiotics in Yen So were OFL, which accounted for over half total concentration of targeted compounds in sediment, whereas CIP contributed highest percentage from 37% to 97% of total antibiotic amount in West Lake. Because concentrations of OFL were significantly higher than other antibiotics obtained in Yen So Lake, this compound accounted for the highest percentage rate in sediment. Indeed, the greatest pollution level of OFL was found at a concentration up to 4,017 ng/g (total amount: 7,456 ng/g) at site YSL. S11, followed by site YSL. S02 at an amount of 3,717 ng/g for OFL (total amount: 5,896 ng/g). Likewise, it was revealed that CIP was a predominant contributor to the antibiotic profile, which was found at notable levels in sediment not only in West Lake but also Yen So Lake. In comparison, AZM made a small contribution to antibiotic composition from several percent to tens of percent in total antibiotic concentrations.

### 3.3. Sources and Fate of Antibiotics in Surface Water and Sediment

Because antibiotics are partially metabolized and excreted, a large amount of these compounds potentially enter wastewater stream through urban rivers prior to accumulate in sediment. Occurrence pattern of antibiotics in sediment suggested that these compounds probably have similar sources being released from anthropogenic activities in urban areas [55] despite their different concentrations. As aforementioned, at a number of sites in Yen So Lake, levels of antibiotics in surface water from the river system were markedly higher than those in the lake system, implying that urban river input was an important contributor of compounds in the lakes. It is noteworthy that the occurrence of targeted compound could be influenced by point-contamination sources and nonpoint sources including elimination of untreated/treated sewage, combination of sewer overflows, and urban stormwater runoff [8]. In the middle of Hanoi capital with very high population density (about 2,300 people/km^2^) [56], two investigated locations suffer from serious municipal wastewater resulting in anthropogenic activities. In fact, inappropriate use of antibiotics is common practice in many countries consisting of Vietnam where these compounds are easily delivered for self-limiting upper respiratory tract diseases without any prescriptions [46]. Therefore, the findings of current study would suggest that households play an important role in discharge of antibiotics to the waste stream. On the other hand, urban areas of Hanoi currently have about 40 public hospitals and countless private hospitals as well as medical centers. Remarkably, there has been not any specific regulations on concentration of antibiotics in environmental compartments in Vietnam, which raises concerns for incomplete removal of antibiotics from common wastewater treatment systems. According to the report from Vietnam's Ministry of Health in 2019, five antibiotics which were often prescribed in hospitals consisted of AMP, CFX, CIP, MXF, and CLR. Among these compounds, AMP with more than 60% of total usage was the most commonly used antibiotic in hospitals, which is similar to the occurrence patterns found in this study. Likewise, the contamination profile of MXF was probably associated with its current status with only 1.4% of total usage [57]. In addition to potential amounts of antibiotics released from hospital effluents [16, 17], abundance of SMX and TMP in this study implied that there is a certain amount of use in fish and shrimp aquaculture in two urban lakes due to the fact that these antibiotics are used in postlarvae to adult stage in Vietnamese shrimp farming [17]. Besides, West Lake and Yen So Lake often receive stormwater runoff from surrounding residential areas, which means unexpected solid waste such as feces from kinds of pet animals tend to be washed into the aquatic environment. Although levels of antibiotics generated from these sources were probably negligible, presence of veterinary antibiotics has previously been recognized in the literature [9, 52].

The fate and transport of antibiotics depend on their physicochemical properties [58] and environmental conditions [27, 49] through their natural attenuation [58]. In fact, different compounds possess different physicochemical properties, such as octanol-water partition coefficient (K_ow_), sediment-water distribution coefficient (Kd), and their solubility in water [59]. For instance, low log K_ow_ of antibiotics (<2.5) revealed low sorption capacity of these compounds that makes them tend to be present in surface water. By contrast, antibiotics with a high molecular weight and high log K_ow_ of >5 are strongly sorbed to sediment [58]. In natural environment, sunlight is considered as major factors to transform pharmaceuticals including antibiotics in the aquatic system. The photodegradation may be accelerated under other conditions, e.g., pH, temperature, total organic carbon (TOC), and metal cations [60]. In the earlier study, Timm et al. [49] proved that the degradation of *β*-lactams might be influenced by photodegradation together with hydrolysis and microorganism activities. It is notable that these compounds are easily degraded by sunlight (1 kW/m^2^) with a short half-live from 3.2 to 7.0 h [49]. Consequently, *β*-lactam antibiotics seem to be negligible in surface water and sediment not only in this study but also in other studies around the world. Apart from the photolytic degradation, the biodegradation occurs by enzymatic reactions, which plays an important role in transformation process of antibiotics in the environment although it might take longer time for biodegradation, especially in anaerobic condition [59]. For example, there are a lot of microbial species to transform and degrade antibiotics, in which *Bacillus sp.*, *Enterobacter sp.*, and *Lactobacillus gasseri* degraded 74, 96, and 100% of CIP (initial level: 5 mg/L) after 14 days [61]. Generally, quinolones are illustrated to recalcitrant to biodegradation in water and sediment [62], depending on microbial distribution and profile. That might be the reason why these compounds were often detected at different pollution levels in the same reservoir in this study. On the other hand, in sediment phase, the microbial degradation tends to be more active than those in aqueous phase due to the fact that microorganisms are vital to the biodegradation activities [59]. Additionally, hydrological factors in the lakes may impact the antibiotic fate in water and sediment [63]. However, it is hard to determine combination of the hydrological and chemical factors in the fate and transport of antibiotics in water-sediment phase [13].

### 3.4. Sediment-Water Distribution Coefficient of Antibiotics

To better understand the dynamics of antibiotic levels between sediment and water, Kd of antibiotics is calculated as the fraction of antibiotic concentrations in sediment and their corresponding concentrations in water [52]. Generally, the Kd values of the antibiotics are important to evaluate their sorption characteristics of individual compound in the aquatic system although these values are not constant [10]. It can be seen that the Kd values were highly valuable in two urban lakes. For example, the Kd values ranged from 124.5 to 61,900 L/kg for OFL, from 9,469 to 536,000 L/kg for CIP, from 11.59 to 1,787 L/kg for MXF, and from 53,100 to 544,000 L/kg for NOR. The compounds which had lower Kd values were SMX (27.87–256.5 L/kg), CLR (27.7–584.4 L/kg), AZM (3,700–114,000 L/kg), and TMP (42.21–552.2 L/kg). While quinolones showed significantly higher Kd values (average value: 104,000 L/kg), three antibiotic classes including macrolides, sulfonamides, and trimethoprim had lower Kd values with an average value of 14,400, 160, and 218.2 L/kg, respectively. The Kd values obtained in the current study are much greater than those calculated in other catchments [11, 14, 26] due to high concentrations of antibiotics in sediment of this report. However, there is a relative similarity in decrease orders of the Kd values as follows: quinolones > macrolides > sulfonamides, which implies strong adsorption of the quinolones onto sediments. In other words, these compounds accumulate more easily in sediment in comparison with other antibiotic categories. Indeed, quinolones were the major compounds in sediments and the levels were many orders of magnitude greater than those in water phase. To assess sediment-water interactions of targeted compounds, the organic carbon concentration in solid phase is a meaningful parameter although there are different levels of sediment-water interactions in the catchment because of multiple conditions (e.g., physicochemical properties, hydrological conditions, and mineral factor). Therefore, if the environmental factors vary, compounds adsorbed by the sediment phase will be discharged into water again [13].

### 3.5. Ecological Risk Assessment

**Figure 4:**
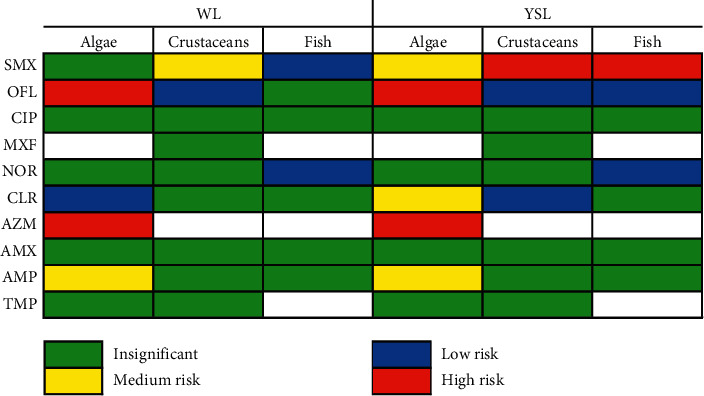
Ecological risks of ten antibiotics in West Lake and Yen So Lake.

Due to the fact that there are limited toxicity data of antibiotics in sediment, especially results related to benthic organisms which directly suffer from negative impact of contamination by antibiotics in sediment, the comprehensive ecological risk of these compounds was only assessed in aquatic ecosystems. The NOEC and EC50 of aquatic organisms including primary consumers (e.g., algae), secondary consumers (e.g., crustaceans), and tertiary consumers (e.g., fish) in the food chain were obtained from the previous literature as shown in Table S5 in the Supplementary Information. Then, the predicted no-effect concentration (PNEC) of antibiotics was calculated by the lowest value of NOEC or EC50 as summarized in Table S6 (Supplementary Materials). From obtained data, the RQ values of investigated compounds in urban lakes are presented in Figure 4. Notably, compared to fish or high level consumers, algae had lower NOEC and EC50, implying that these species are more sensitive to selected antibiotics [64].

Obviously, the RQ values of OFL for algae were higher than 1, suggesting that these compounds were harmful to algae in the lakes, which is consistent with the results reported in Zizhuyuan Lake [65], Hanjiang River [10], and Chaohu Lake [66]. In addition, AZM could cause high risks to algae, which may be due to their higher concentrations and lower PNEC values. As for SMX, the RQ value was found to be greater 1 in Yen So Lake for both crustaceans (*Ceriodaphnia dubia*) and fish (*Carassius auratus*), implying that this compound may pose high ecological risks to freshwater organisms. It is noteworthy that each of these organisms was the representative of different consumers, which reflected their potential to bioaccumulate toxic substances through the food web. Meanwhile, CIP, MXF, AMP, NOR, AMX, and TMP are not likely at risk to algae because their risk quotients are much less than 0.1. In comparison with other results in different lakes and rivers, RQs of antibiotics are similar to those in Zizhuyuan Lake [65] and Dongjiang River in China [13]. In association with multiple impacts of antibiotic accumulation to aquatic organisms, antibiotic resistance genes (ARGs) have gained increasing attention, which should be given priority controls. The evolution and dissemination of ARGs which have been recognized in the previous literature revealed their potential adverse effect on aquatic ecosystems and public health [67]. For this reason, it is vital to evaluate antibiotic pollution in West Lake as well as Yen So Lake, which is urgently needed to control their ecological risks because aquaculture activities of these lakes have usually occurred to provide numerous amount of fish to urban markets in the city.

## 4. Conclusions

The data in the current study showed that abundance of these antibiotics in water was consistent whereas levels in sediment were much higher than other studies around the world. Among fifteen antibiotics, 12 targeted compounds were detected in surface water while only eight antibiotics belonging to 4 classes were observed in sediment, except for *β*-lactam. The obtained results suggested wide usage patterns of individuals in urban areas where households and hospitals might be important sources of antibiotics to the waste stream due to application of human medicine and incomplete removal during sewage treatment. Depending on their physicochemical characteristics and environmental conditions, the fate and transportation of antibiotics differed in water and sediment. Notably, both of lakes hold high ecological risks of OFL and AZM and medium risks of several antibiotics. Therefore, comprehensive attention should be paid to monitor contamination by ARGs and ARBs released from urban lakes and rivers in terms of the safety control of water quality in the near future.

## Figures and Tables

**Table 1:** Optimized UPLC-ESI-MS/MS parameters for selected antibiotics.

Antibiotics	ESI mode	Retention time (min)	Precursor ion (m/*z*)	Product ions (m/*z*)	Quantitive ion (m/*z*)	Q1 prebias (V)	Ce	Q3 prebias (V)
SMX	Positive	10.21	253.95	92.15/156.05	92.15	10	29	10
OFL	Positive	8.59	361.95	318.10/261.05	318.1	14	20	21
CIP	Positive	8.88	332	314.1/230.8	314.1	24	22	23
MXF	Positive	12.1	402	384.05/358.2	384.05	30	22	27
NOR	Positive	8.65	320.05	302.2/276.1	302.2	16	21	30
CLR	Positive	19.49	748.25	158/590.2	158	28	32	20
AZM	Positive	13.98	752.4	594.3	594.3	28	32	28
CFM	Positive	7.88	454.7	285.3/126.2	126.2	14	30	22
CTX	Positive	8.17	455.9	396.05/124.95	396.05	17	11	20
CEC	Positive	7.06	367.95	106.1/173.95	106.1	18	25	21
CFX	Positive	7.9	347.95	158/173.95	158	13	10	18
CDX	Positive	5.13	364	114/208.05	114	14	11	22
AMX	Positive	3.91	366.05	349.05/114	349.05	19	10	25
AMP	Positive	7.89	350.15	106.05/159.95	106.05	18	13	17
TMP	Positive	8.15	291.05	230.1/261.05	230.1	22	26	28

**Table 2:** Instrument performance and validation data.

Antibiotics	IDL (ng/mL)	IQL (ng/mL)	*R* ^2^	Linear range (ng/mL)
SMX	0.26	0.86	0.999	1–500
CIP	1.18	3.93	0.997	5–1000
OFL	1.05	3.51	0.996	5–1500
MXF	1.45	4.85	0.998	5–500
NOR	1.72	5.74	0.999	10–500
CLR	0.08	0.26	0.999	0.5–500
AZM	0.35	1.16	0.999	2–500
CFM	2.31	7.69	0.999	10–2000
CTX	1.73	5.78	0.999	10–500
CEC	2.28	7.59	0.999	10–1000
CFX	2.03	6.78	0.999	10–500
CDX	2.01	6.69	0.999	10–2000
AMP	2.45	8.18	0.999	10–1000
AMX	2.65	8.85	0.999	10–1000
TMP	0.07	0.22	0.999	0.5–500

**Table 3:** Concentrations of antibiotics detected in surface water from two lakes in comparison with other rivers and lakes in other studies.

Class	Antibiotics	In this study				In other studies		
		West Lake (*n* = 10)		Yen So Lake (*n* = 14)				
		Range (ng/L)	Mean (ng/L)	Range (ng/L)	Mean (ng/L)	Range (ng/L)	Region	References
SAs	SMX	4.54–25.04	7.51	12.86–806.5	178.9	<LOD-652.7	Spain	[25]
						3.6–1,435	France	[26]
						1.12–13.28	China	[27]
						108–3,508	Vietnam	[8]
						<LOD-480	Germany	[28]

QNs	OFL	28.18–40.9	33.20	26.96–158.7	73.62	12–360	Latin America	[29]
						74.6–308.4	China	[30]
						8–1,904	Spain	[31]
	CIP	0.23–3.37	0.72	<LOD-6.05	1.68	<LOD-38.1	China	[30]
						<LOD-1.3	Australia	[32]
						<LOD-2.5	Brazil	[6]
	MXF	<LOD	<LOD	<LOD-20.44	11.56	34–72	China	[33]
	NOR	<LOD-0.52	0.19	<LOD-0.64	0.07	<LOD-38	Pakistan	[34]
						51	Brazil	[35]
						<LOD-210	Australia	[36]

MLs	CLR	<LOD-9.78	1.75	7.23–243	45.76	4–65	Vietnam	[8]
						0.5–130	Pakistan	[34]
						<LOD-260	Germany	[28]
	AZM	<LOD-3.28	0.56	<LOD-20.54	1.73	0.2–79.2	Singapore	[37]
						165–233.3	Iran	[38]
						<LOD-115.5	Spain	[39]

*β*-Ls	CFM	<LOD-575.4	57.54	<LOD-1572	87.35	278.7–422.1	Iran	[38]
						<LOD	Vietnam	[8]
	CTX	<LOD-47.16	8.93	<LOD-84.21	33.05	<LOD-0.82	China	[40]
	CEC	<LOD-301.3	51.67	<LOD-308.2	48.59	<LOD-200	Australia	[32]
						<LOD-0.97	China	[40]

*β*-Ls	CFX	<LOD-0.68	0.23	<LOD-19.09	2.03	<LOD-133	Brazil	[6]
						122–460	Iran	[38]
						26.8–2,000	Australia	[36]
	CDX	<LOD	<LOD	<LOD-1.52	0.10	<LOD-18.25	Egypt	[41]
	AMX	<LOD-221.3	51.83	<LOD-104	17.36	<LOD-1,126	Vietnam	[8]
						63–97.4	Iran	[38]
						<LOD-2.7	Ghana	[42]
						1.9–25.2	Italy	[43]
	AMP	<LOD-70.14	39.09	<LOD-81.76	28.49	21–184	Ghana	[42]
						<LOD	Vietnam	[8]

TMs	TMP	0.65–3.14	1.11	6.55–67.90	13.27	<LOD-92.7	Spain	[39]
						<LOD-130	Australia	[32]
						2.4–252	UK	[5]
						0.4–1,700	Pakistan	[34]
						17–820	Ghana	[42]

*Abbreviations*. n: number of collected samples; LOD: limit of detection.

## Data Availability

The data and supplementary materials used to support the results of this study are included within the article.

## Supplementary Materials

Supplementary Materials Table S1. Concentration of antibiotics (ng/L) in surface water samples in Westlake Table S2. Concentration of antibiotics (ng/L) in surface water samples in Yen So Lake Table S3. Concentration of antibiotics (ng/g) in sediment samples in Westlake Table S4. Concentration of antibiotics (ng/g) in sediment samples in Yen So Lake Table S5. Summary on freshwater aquatic toxicity data of antibiotics Table S6. Summary of antibiotics' predicted no effect concentration (PNEC) by NOEC or EC50.
