# Supplementary material for: Occurrence, Distribution, and Ecological Risk Assessment of Antibiotics in Selected Urban Lakes of Hanoi, Vietnam
Source: J Anal Methods Chem. 2021 Mar 10;2021:6631797. doi: 10.1155/2021/6631797 (PMC7972860; doi:10.1155/2021/6631797)
Supplement: Supplementary Materials — Table S1. Concentration of antibiotics (ng/L) in surface water samples in Westlake Table S2. Concentration of antibiotics (ng/L) in surface water samples in Yen So Lake Table S3. Concentration of antibiotics (ng/g) in sediment samples in Westlake Table S4. Concentration of antibiotics (ng/g) in sediment samples in Yen So Lake Table S5. Summary on freshwater aquatic toxicity data of antibiotics Table S6. Summary of antibiotics' predicted no effect concentration (PNEC) by NOEC or EC50. [file 6631797.f1.docx]

**Supplementary Information**

**Occurrence, distribution and ecological risk assessment of antibiotics in selected urban lakes of Hanoi, Vietnam**

Hong Anh Duong,^1^ Thi Vi Phung,^2^ Thuy Ngoc Nguyen,^1^ Thi Lan-Anh Phan,^2^ and Hung Viet Pham^1,2^

*^1^ Research Centre for Environmental Technology and Sustainable Development, VNU University of Science, Vietnam National University, Hanoi, 334 Nguyen Trai, Thanh Xuan, Hanoi 10000, Vietnam
^2^ Key Laboratory of Analytical Technology for Environmental Quality and Food Safety Control, VNU University of Science, Vietnam National University, Hanoi, 334 Nguyen Trai, Thanh Xuan, Hanoi 100000, Vietnam*

Correspondence should be addressed to Hung Viet Pham; phamhungviet@hus.edu.vn

**Table S1.** Concentration of antibiotics (ng/L) in surface water samples in Westlake

**Table S2.** Concentration of antibiotics (ng/L) in surface water samples in Yen So Lake

**Table S3.** Concentration of antibiotics (ng/g) in sediment samples in Westlake

**Table S4.** Concentration of antibiotics (ng/g) in sediment samples in Yen So Lake

**Table S5.** Summary on freshwater aquatic toxicity data of antibiotics

**Table S6.** Summary of antibiotics’ predicted no effect concentration (PNEC) by NOEC or EC50

| **No.** | **Class** | **Name** | **WL.W01** | **WL.W02** | **WL.W03** | **WL.W04** | **WL.W05** | **WL.W06** | **WL.W07** | **WL.W08** | **WL.W09** | **WL.W10** | **Min** | **Max** | **Average** |
| --- | --- | --- | --- | --- | --- | --- | --- | --- | --- | --- | --- | --- | --- | --- | --- |
| 1 | SAs | SMX | 5.56 | 6.03 | 6.35 | 5.25 | 7.15 | 4.54 | 5.65 | 4.85 | 25.04 | 4.67 | 4.54 | 25.04 | 7.51 |
| 2 | QNs | OFL | 31.09 | 40.90 | 39.61 | 28.51 | 32.31 | 28.39 | 28.18 | 30.44 | 39.96 | 32.65 | 28.18 | 40.90 | 33.20 |
| 3 |  | CIP | 0.54 | 0.70 | 0.37 | 0.28 | 0.26 | 0.45 | 0.40 | 0.23 | 3.37 | 0.58 | 0.23 | 3.37 | 0.72 |
| 4 |  | MXF | < LOD | < LOD | < LOD | < LOD | < LOD | < LOD | < LOD | < LOD | < LOD | < LOD | < LOD | < LOD | < LOD |
| 5 |  | NOR | < LOD | 0.52 | < LOD | < LOD | < LOD | < LOD | 0.16 | 0.09 | 0.34 | 0.25 | < LOD | 0.52 | 0.19 |
| 6 | MLs | CLR | 0.94 | 1.03 | < LOD | 0.83 | 1.01 | 0.73 | 1.06 | 1.05 | 9.78 | 1.10 | < LOD | 9.78 | 1.75 |
| 7 |  | AZM | < LOD | < LOD | < LOD | 0.27 | < LOD | < LOD | 0.21 | < LOD | < LOD | 0.13 | < LOD | 3.28 | 0.56 |
| 8 | β-Ls | CFM | < LOD | < LOD | < LOD | 575.36 | < LOD | < LOD | < LOD | < LOD | < LOD | < LOD | < LOD | 575.36 | 57.54 |
| 9 |  | CTX | 47.16 | < LOD | < LOD | < LOD | < LOD | < LOD | 42.18 | < LOD | < LOD | < LOD | < LOD | 47.16 | 8.93 |
| 10 |  | CEC | < LOD | < LOD | 301.28 | < LOD | < LOD | 215.38 | < LOD | < LOD | < LOD | < LOD | < LOD | 301.28 | 51.67 |
| 11 |  | CFX | 0.68 | < LOD | < LOD | < LOD | < LOD | < LOD | < LOD | < LOD | < LOD | < LOD | < LOD | 0.68 | 0.23 |
| 12 |  | CDX | < LOD | < LOD | < LOD | < LOD | < LOD | < LOD | < LOD | < LOD | < LOD | < LOD | < LOD | < LOD | < LOD |
| 13 |  | AMX | < LOD | < LOD | 221.26 | < LOD | < LOD | 157.80 | < LOD | 139.28 | < LOD | < LOD | < LOD | 221.26 | 51.83 |
| 14 |  | AMP | 67.72 | < LOD | < LOD | 62.66 | 70.14 | < LOD | 61.24 | 65.92 | 63.18 | < LOD | < LOD | 70.14 | 39.09 |
| 15 | TMP | TMP | 0.85 | 0.89 | 0.98 | 0.89 | 0.78 | 0.65 | 1.29 | 0.71 | 3.14 | 0.94 | 0.65 | 3.14 | 1.11 |
| Total: | | | 154.55 | 50.07 | 569.84 | 674.05 | 111.65 | 407.94 | 140.37 | 242.56 | 148.09 | 40.32 | 33.60 | 1301.91 | 254.33 |

**Table S1.** Concentration of antibiotics (ng/L) in surface water samples in Westlake.

*LOD: Limit of Detection

**Table S2.** Concentration of antibiotics (ng/L) in surface water samples in Yen So Lake.

| Class | Name | YS. W01 | YS. W02 | YS. W03 | YS. W04 | YS. W05 | YS. W06 | YS. W07 | YS. W08 | YS. W09 | YS. W10 | YS. W11 | YS. W12 | YS. W13 | YS. W14 | YS. W11(SR) | YS. W12(SR) | YS. W13(SR) | YS. W14(SR) | Min | Max | Average |
| --- | --- | --- | --- | --- | --- | --- | --- | --- | --- | --- | --- | --- | --- | --- | --- | --- | --- | --- | --- | --- | --- | --- |
| SAs | SMX | 111.32 | 135.19 | 135.09 | 26.99 | 33.56 | 12.86 | 23.14 | 26.69 | 40.57 | 272.88 | 60.80 | 24.70 | 599.37 | 21.95 | 269.86 | 158.77 | 459.88 | 806.49 | 12.86 | 806.49 | 201.02 |
| QNs | OFL | 79.53 | 78.81 | 75.94 | 55.14 | 58.53 | 37.85 | 37.08 | 26.96 | 29.89 | 101.86 | 64.91 | 38.74 | 129.71 | 55.83 | 111.60 | 96.12 | 158.68 | 87.96 | 26.96 | 158.68 | 76.57 |
|  | CIP | 6.05 | 5.56 | 4.61 | 4.56 | 3.67 | < LOD | < LOD | < LOD | < LOD | < LOD | < LOD | < LOD | < LOD | < LOD | < LOD | < LOD | 5.73 | < LOD | < LOD | 6.05 | 1.91 |
|  | MXF | 12.36 | 13.81 | 13.30 | 12.47 | < LOD | < LOD | 12.06 | 12.13 | 12.33 | 13.53 | 12.78 | < LOD | 14.71 | 13.67 | 14.40 | 16.29 | 13.75 | 20.44 | < LOD | 20.44 | 11.72 |
|  | NOR | 0.17 | 0.25 | 0.12 | 0.64 |  | < LOD | < LOD | < LOD | < LOD | < LOD | < LOD | < LOD | < LOD | < LOD | < LOD | < LOD | < LOD | < LOD | < LOD | 0.64 | 0.10 |
| MLs | CLR | 26.98 | 26.23 | 25.07 | 17.13 | 18.33 | 7.23 | 19.77 | 11.83 | 9.62 | 28.42 | 51.26 | 22.86 | 115.32 | 35.95 | 64.40 | 66.98 | 33.16 | 243.04 | 7.23 | 243.04 | 49.42 |
|  | AZM |  |  | 0.57 |  |  | < LOD | < LOD | < LOD | < LOD | < LOD | < LOD | 1.02 | < LOD | 2.04 | < LOD | 20.54 | < LOD | < LOD | < LOD | 20.54 | 1.75 |
| β-Ls | CFM | < LOD | < LOD | < LOD | < LOD | < LOD | < LOD | < LOD | 1572.35 | < LOD | < LOD | < LOD | < LOD | < LOD | < LOD | < LOD | < LOD | < LOD | < LOD | < LOD | 1572.35 | 165.51 |
|  | CTX | 52.86 | 58.37 | 49.63 | 48.80 | < LOD | 48.38 | 48.14 | 46.79 | < LOD | 56.17 | < LOD | < LOD | < LOD | < LOD | 53.63 | < LOD | 47.99 | 84.21 | < LOD | 84.21 | 34.39 |
|  | CEC | < LOD | < LOD | < LOD | 285.15 | < LOD | < LOD | < LOD | < LOD | < LOD | < LOD | < LOD | < LOD | 308.21 | < LOD | 281.21 | < LOD | < LOD | < LOD | < LOD | 308.21 | 62.25 |
|  | CFX |  |  |  |  |  | 0.80 | 3.33 | < LOD | 1.90 | < LOD | < LOD | < LOD | < LOD | < LOD | 0.45 | < LOD | < LOD | 19.90 | < LOD | 19.90 | 2.12 |
|  | CDX | < LOD | < LOD |  |  |  | < LOD | 1.52 | < LOD | < LOD | < LOD | < LOD | < LOD | < LOD | < LOD | 0.02 | < LOD | < LOD | < LOD | < LOD | 1.52 | 0.19 |
|  | AMX | 65.47 | < LOD | < LOD | < LOD | 104.02 | < LOD | < LOD | < LOD | < LOD | < LOD | 66.20 | < LOD | 76.74 | < LOD | < LOD | < LOD | < LOD | < LOD | < LOD | 104.02 | 21.92 |
|  | AMP | 44.18 | 45.14 | < LOD | < LOD | 47.71 | < LOD | 44.55 | 48.14 | < LOD | < LOD | 47.39 | < LOD | 48.95 | < LOD | < LOD | 59.34 | 45.63 | 81.76 | < LOD | 81.76 | 29.57 |
| TMP | TMP | 6.75 | 6.62 | 6.55 | 6.57 | 7.48 | 7.41 | 10.65 | 7.91 | 7.82 | 7.15 | 21.31 | 13.72 | 17.45 | 14.63 | 7.67 | 9.88 | 11.38 | 67.90 | 6.55 | 67.90 | 13.69 |
| Total: | | 405.68 | 369.97 | 310.88 | 457.46 | 273.32 | 114.52 | 200.25 | 1752.80 | 102.14 | 480.03 | 324.66 | 101.03 | 1310.48 | 144.07 | 803.23 | 427.92 | 776.21 | 1411.70 |  |  |  |

* LOD: Limit of Detection

**Table S3.** Concentration of antibiotics (ng/g) in sediment samples in Westlake.

| Class |  | WL.S01 | WL.S02 | WL.S03 | WL.S04 | WL.S05 | WL.S06 | WL.S07 | WL.S08 | WL.S09 | WL.S10 | WL.S11 | Min | Max | Average |
| --- | --- | --- | --- | --- | --- | --- | --- | --- | --- | --- | --- | --- | --- | --- | --- |
| SAs | SMX | < LOD | < LOD | 1.18 | 1.35 | < LOD | < LOD | < LOD | < LOD | < LOD | < LOD | < LOD | < LOD | 1.35 | 0.23 |
| QNs | OFL | < LOD | < LOD | < LOD | < LOD | < LOD | < LOD | 3.51 | 113.93 | 5.44 | 7.51 | < LOD | 3.51 | 113.93 | 32.60 |
|  | CIP | 25.30 | 29.37 | 12.79 | 16.84 | 22.34 | 6.82 | 24.54 | 121.05 | 31.91 | 30.91 | 29.11 | 6.82 | 121.05 | 31.91 |
|  | MXF | < LOD | < LOD | < LOD | < LOD | < LOD | < LOD | < LOD | < LOD | < LOD | < LOD | < LOD | < LOD | < LOD | < LOD |
|  | NOR | 20.86 | < LOD | 13.53 | 16.06 | 24.98 | 7.94 | 23.64 | 48.25 | 17.96 | 20.68 | 10.50 | < LOD | 48.25 | 18.58 |
| MLs | CLR | 0.38 | 0.45 | 0.36 | 0.42 | 0.65 | 0.23 | 0.58 | 0.79 | 0.52 | 0.59 | 0.34 | 0.23 | 0.79 | 0.48 |
|  | AZM | < LOD | < LOD | 6.37 | 6.05 | 8.40 | 3.03 | 12.25 | 29.63 | 15.44 | 14.70 | 11.56 | < LOD | 29.63 | 9.77 |
| β-Ls | CFM | < LOD | < LOD | < LOD | < LOD | < LOD | < LOD | < LOD | < LOD | < LOD | < LOD | < LOD | < LOD | < LOD | < LOD |
|  | CTX | < LOD | < LOD | < LOD | < LOD | < LOD | < LOD | < LOD | < LOD | < LOD | < LOD | < LOD | < LOD | < LOD | < LOD |
|  | CEC | < LOD | < LOD | < LOD | < LOD | < LOD | < LOD | < LOD | < LOD | < LOD | < LOD | < LOD | < LOD | < LOD | < LOD |
|  | CFX | < LOD | < LOD | < LOD | < LOD | < LOD | < LOD | < LOD | < LOD | < LOD | < LOD | < LOD | < LOD | < LOD | < LOD |
|  | CDX | < LOD | < LOD | < LOD | < LOD | < LOD | < LOD | < LOD | < LOD | < LOD | < LOD | < LOD | < LOD | < LOD | < LOD |
|  | AMX | < LOD | < LOD | < LOD | < LOD | < LOD | < LOD | < LOD | < LOD | < LOD | < LOD | < LOD | < LOD | < LOD | < LOD |
|  | AMP | < LOD | < LOD | < LOD | < LOD | < LOD | < LOD | < LOD | < LOD | < LOD | < LOD | < LOD | < LOD | < LOD | < LOD |
| TMP | TMP | < LOD | 0.49 | < LOD | < LOD | < LOD | < LOD | < LOD | < LOD | < LOD | < LOD | 0.26 | < LOD | 0.49 | 0.07 |
| Total: | | 46.53 | 30.30 | 34.23 | 40.73 | 56.36 | 18.02 | 64.52 | 313.65 | 71.27 | 74.38 | 51.77 | < LOD | < LOD | < LOD |

LOD: Limit of Detection

**Table S4.** Concentration of antibiotics (ng/g) in sediment samples in Yen So Lake.

| Class |  | YSL.S01 | YSL.S02 | YSL. S03 | YSL. S04 | YSL. S05 | YSL. S06 | YSL. S07 | YSL.S08 | YSL.S09 | YSL.S10 | YSL.S11 | YSL.S12 | YSL.S13 | YSL.S14 | Min | Max | Average |
| --- | --- | --- | --- | --- | --- | --- | --- | --- | --- | --- | --- | --- | --- | --- | --- | --- | --- | --- |
| SAs | SMX | < LOD | 3.77 | < LOD | < LOD | < LOD | 2.18 | < LOD | < LOD | < LOD | < LOD | 2.36 | < LOD | < LOD | < LOD | < LOD | 3.77 | 0.59 |
| QNs | OFL | 1271.38 | 3716.73 | 572.81 | 2089.86 | 2031.86 | 1777.49 | 626.21 | 816.03 | 411.26 | 281.36 | 4016.97 | 504.87 | 295.93 |  | 281.36 | 4016.97 | 1416.37 |
|  | CIP | 562.26 | 1797.46 | 151.55 | 1157.61 | 1126.42 | 1016.61 | 281.26 | 191.79 | 109.04 | 221.26 | 2368.37 | 230.69 | 117.81 | 26.78 | 26.78 | 2368.37 | 668.49 |
|  | MXF | 7.11 | 24.68 | 1.34 | 12.59 | 11.88 | 19.42 |  | 0.14 |  |  | 55.24 | 4.76 |  |  | 0.14 | 55.24 | 15.24 |
|  | NOR | 53.62 | 94.05 | 48.47 | 124.93 | 121.56 | 66.76 | 50.53 | 48.20 | 47.38 | 18.04 | 38.89 | 40.48 | 28.40 | 21.15 | 18.04 | 124.93 | 57.32 |
| MLs | CLR | 0.75 | 2.26 | < LOD | 1.25 | 1.21 | 1.33 | < LOD | 0.79 | 0.85 | 2.56 | 4.33 | 1.51 | 0.91 | 1.24 | < LOD | 4.33 | 1.36 |
|  | AZM | 23.95 | 256.16 | 26.19 | 227.61 | 221.48 | 226.37 | 40.98 | 59.51 | 53.50 | 86.90 | 969.18 | 116.24 | 63.30 | 7.56 | 7.56 | 969.18 | 169.92 |
| β-Ls | CFM | < LOD | < LOD | < LOD | < LOD | < LOD | < LOD | < LOD | < LOD | < LOD | < LOD | < LOD | < LOD | < LOD | < LOD | < LOD | < LOD | < LOD |
|  | CTX | < LOD | < LOD | < LOD | < LOD | < LOD | < LOD | < LOD | < LOD | < LOD | < LOD | < LOD | < LOD | < LOD | < LOD | < LOD | < LOD | < LOD |
|  | CEC | < LOD | < LOD | < LOD | < LOD | < LOD | < LOD | < LOD | < LOD | < LOD | < LOD | < LOD | < LOD | < LOD | < LOD | < LOD | < LOD | < LOD |
|  | CFX | < LOD | < LOD | < LOD | < LOD | < LOD | < LOD | < LOD | < LOD | < LOD | < LOD | < LOD | < LOD | < LOD | < LOD | < LOD | < LOD | < LOD |
|  | CDX | < LOD | < LOD | < LOD | < LOD | < LOD | < LOD | < LOD | < LOD | < LOD | < LOD | < LOD | < LOD | < LOD | < LOD | < LOD | < LOD | < LOD |
|  | AMX | < LOD | < LOD | < LOD | < LOD | < LOD | < LOD | < LOD | < LOD | < LOD | < LOD | < LOD | < LOD | < LOD | < LOD | < LOD | < LOD | < LOD |
|  | AMP | < LOD | < LOD | < LOD | < LOD | < LOD | < LOD | < LOD | < LOD | < LOD | < LOD | < LOD | < LOD | < LOD | < LOD | < LOD | < LOD | < LOD |
| TMP | TMP | < LOD | 1.23 | < LOD | < LOD | < LOD | 0.71 | < LOD | < LOD | < LOD | < LOD | 0.90 | 0.73 | < LOD | 0.57 | < LOD | 1.23 | 0.30 |
| Total: | | 1919.08 | 5896.34 | 800.36 | 3613.84 | 3514.41 | 3110.86 | 998.97 | 1116.46 | 622.01 | 610.12 | 7456.24 | 899.27 | 506.36 | 57.30 |  |  |  |

LOD: Limit of Detection

**Table S5.** Summary on freshwater aquatic toxicity data of antibiotics.

| Antibiotics | CAS No. | Class | Species | Endpoint | Value  (mg/L) | Reference |
| --- | --- | --- | --- | --- | --- | --- |
| Sulfamethoxazole | 723466 | Algae | *Pseudokirchneriella subcapitata* | NOEC | 0.5 | [1] |
|  |  |  | *Microcystis aeruginosa* | NOEC | 0.55 | [2] |
|  |  |  | *Chlorella fusca var. vacuolata* | NOEC | 1.54 | [3] |
|  |  | Crustaceans | *Ceriodaphnia dubia* | EC50 | 0.21 | [4] |
|  |  |  | *Daphnia magna* | EC50 | 25.2 | [4] |
|  |  |  | *Moina macrocopa* | EC50 | 70.4 | [5] |
|  |  | Fish | *Carassius auratus* | NOEC | 0.08 | [6] |
|  |  |  | *Danio rerio* | NOEC | 0.533 | [7] |
|  |  |  | *Oncorhynchus mykiss* | NOEC | 7.9148625 | [8] |
| Ofloxacin | 82419361 | Algae | *Pseudokirchneriella subcapitata* | EC50 | 1.44 | [4] |
|  |  |  | *Microcystis aeruginosa* | EC50 | 0.021 | [9] |
|  |  | Crustaceans | *Ceriodaphnia dubia* | EC50 | 3.13 | [4] |
|  |  |  | *Daphnia magna* | EC50 | 31.75 | [4] |
|  |  |  | *Daphnia magna* | NOEC | 10 | [9] |
|  |  | Fish | *Pimephales promelas* | NOEC | 10 | [9] |
| Ciprofloxacin | 85721331 | Algae | *Pseudokirchneriella subcapitata* | NOEC | 0.5 | [1] |
|  |  | Crustaceans | *Daphnia magna* | EC50 | 1 | [10] |
|  |  | Fish | *Oncorhynchus mykiss* | NOEC | 3.313466 | [11] |
| Moxifloxacin | 151096092 | Crustaceans | *Daphnia magna* | EC50 | 7 | [12] |
|  |  |  | *Ceriodaphnia dubia* | EC50 | 26 | [12] |
| Norfloxacin | 70458967 | Algae | *Pseudokirchneriella subcapitata* | EC50 | 16.6 | [13] |
|  |  |  | *Anabaena cylindrica* | EC50 | 0.053 | [14] |
|  |  |  | *Synechococcus leopoliensis* | EC50 | 0.63 | [14] |
|  |  | Crustaceans | *Daphnia magna* | NOEC | 0.12 | [15] |
|  |  | Fish | *Carassius auratus* | NOEC | 0.0027 | [16] |
| Clarithromycin | 81103119 | Algae | *Pseudokirchneriella subcapitata* | NOEC | 0.04 | [17] |
|  |  | Crustaceans | *Ceriodaphnia dubia* | EC50 | 8.16 | [4] |
|  |  |  | *Daphnia magna* | EC50 | 25.72 | [4] |
|  |  | Fish | *Oryzias latipes* | LC50 | 100 | [10] |
|  |  |  | *Danio rerio* | NOEC | 1000 | [4] |
| Azithromycin | 83905015 | Algae | *Microcystis aeruginosa* | NOEC | 0.00019 | [18] |
|  |  |  | *Green algae (Chlorophyta)* | NOEC | 0.0018 | [18] |
|  |  |  | *Pseudokirchneriella subcapitata* | EC50 | 0.019 | [19] |
|  |  | Crustaceans | *Daphnia magna* | NOEC | 0.0044 | [18] |
|  |  | Fish | *Oncorhynchus mykiss* | EC50 | 84 | [18] |
| Amoxicillin | 26787780 | Algae | *Anabaena sp.* | EC50 | 56.3 | [20] |
|  |  |  | *Pseudokirchneriella subcapitata* | EC50 | 1500 | [20] |
|  |  |  | *Phaeodactylum tricornutum* | NOEC | 250 | [21] |
|  |  | Crustaceans | *Daphnia magna* | EC50 | 1000 | [5] |
|  |  |  | *Moina macrocopa* | EC50 | 1000 | [5] |
|  |  | Fish | *Danio rerio* | EC50 | 132.4 | [22] |
|  |  |  | *Oryzias latipes* | LC50 | 1000 | [5] |
|  |  |  | *Danio rerio* | NOEC | 10 | [22] |
|  |  |  | *Oncorhynchus mykiss* | NOEC | 182.702 | [8] |
| Ampicillin | 69534 | Algae | *Microcystis aeruginosa* | NOEC | 0.01 | [23] |
|  |  |  | *Pseudokirchneriella subcapitata* | EC50 | 2200 | [24] |
|  |  | Crustaceans | *Daphnia magna* | EC50 | 1000 | [5] |
|  |  |  | *Moina macrocopa* | EC50 | 1000 | [5] |
| Trimethoprim | 738705 | Algae | *Anabaena cylindrica* | NOEC | 200 | [14] |
|  |  |  | *Synechococcus leopoliensis* | NOEC | 13 | [14] |
|  |  |  | *Pseudokirchneriella subcapitata* | NOEC | 12.5 | [25] |
|  |  |  | *Microcystis aeruginosa* | NOEC | 100 | [14] |
|  |  | Crustaceans | *Moina macrocopa* | EC50 | 54.8 | [5] |
|  |  |  | *Daphnia magna* | NOEC | 3.12 | [25] |

**Table S6.** Summary of antibiotics’ predicted no effect concentration (PNEC) by NOEC or EC50.

| Antibiotics | Species | Reference | Endpoint | Value  (mg/L) | AF | PNEC (ng/L) |
| --- | --- | --- | --- | --- | --- | --- |
| Sulfamethoxazole | *Pseudokirchneriella subcapitata* | [1] | NOEC | 0.5 | 100 | 5 x 10^3^ |
|  | *Ceriodaphnia dubia* | [4] | EC50 | 0.21 | 1000 | 210 |
|  | *Carassius auratus* | [6] | NOEC | 0.08 | 100 | 800 |
| Ofloxacin | *Microcystis aeruginosa* | [9] | EC50 | 0.021 | 1000 | 21 |
|  | *Ceriodaphnia dubia* | [4] | EC50 | 3.13 | 1000 | 3.13 x 10^3^ |
|  | *Pimephales promelas* | [9] | NOEC | 10 | 1000 | 10^4^ |
| Ciprofloxacin | *Pseudokirchneriella subcapitata* | [1] | NOEC | 0.5 | 100 | 5 x 10^3^ |
|  | *Daphnia magna* | [10] | EC50 | 1 | 1000 | 10^3^ |
|  | *Oncorhynchus mykiss* | [11] | NOEC | 3.313466 | 1000 | 3.31 x 10^3^ |
| Moxifloxacin | *Daphnia magna* | [12] | EC50 | 7 | 1000 | 7 x 10^3^ |
| Norfloxacin | *Pseudokirchneriella subcapitata* | [13] | EC50 | 16.6 | 1000 | 16.6 x 10^3^ |
|  | *Daphnia magna* | [15] | NOEC | 0.12 | 100 | 1.2 x 10^3^ |
|  | *Carassius auratus* | [16] | NOEC | 0.0027 | 100 | 27 |
| Clarithromycin | *Pseudokirchneriella subcapitata* | [17] | NOEC | 0.04 | 100 | 400 |
|  | *Ceriodaphnia dubia* | [4] | EC50 | 8.16 | 1000 | 8.16 x 10^3^ |
|  | *Danio rerio* | [4] | NOEC | 1000 | 100 | 10^7^ |
| Azithromycin | *Microcystis aeruginosa* | [18] | NOEC | 0.00019 | 100 | 1.9 |
| Amoxicillin | *Anabaena sp.* | [20] | EC50 | 56.3 | 1000 | 56.3 x 10^3^ |
|  | *Daphnia magna* | [5] | EC50 | 1000 | 1000 | 10^6^ |
|  | *Danio rerio* | [22] | EC50 | 132.4 | 1000 | 132.4 x 10^3^ |
| Ampicillin | *Microcystis aeruginosa* | [23] | NOEC | 0.01 | 100 | 100 |
|  | *Daphnia magna* | [5] | EC50 | 1000 | 1000 | 10^7^ |
| Trimethoprim | *Anabaena cylindrica* | [14] | NOEC | 200 | 100 | 2 x 10^7^ |
|  | *Moina macrocopa* | [5] | EC50 | 54.8 | 1000 | 54.8 x 10^3^ |

**References**

[1] B. Liu *et al.*, “Growth response and toxic e ff ects of three antibiotics on Selenastrum capricornutum evaluated by photosynthetic rate and chlorophyll biosynthesis,” *J. Environ. Sci.*, vol. 23, no. 9, pp. 1558–1563, 2011.

[2] E. Van Der Grinten, M. G. Pikkemaat, and E. Van Den Brandhof, “Chemosphere Comparing the sensitivity of algal , cyanobacterial and bacterial bioassays to different groups of antibiotics,” *Chemosphere*, vol. 80, no. 1, pp. 1–6, 2010.

[3] S. Stolte *et al.*, “Chemosphere Ecotoxicity evaluation of selected sulfonamides,” vol. 85, pp. 928–933, 2011.

[4] M. Isidori, M. Lavorgna, A. Nardelli, L. Pascarella, and A. Parrella, “Toxic and genotoxic evaluation of six antibiotics on non-target organisms,” vol. 346, pp. 87–98, 2005.

[5] S. Park and Æ. K. Choi, “Park S , Choi K . Hazard assessment of commonly used agricultural antibiotics Hazard assessment of commonly used agricultural antibiotics on aquatic ecosystems,” no. September, 2008.

[6] Z. Li, G. Lu, X. Yang, and C. Wang, “Single and combined effects of selected pharmaceuticals at sublethal concentrations on multiple biomarkers in Carassius auratus,” pp. 353–361, 2012.

[7] T. Vieira, M. João, and C. Cruzeiro, “The toxicity potential of pharmaceuticals found in the Douro River estuary ( Portugal ): Evaluation of impacts on fish liver , by histopathology , stereology , vitellogenin and CYP1A immunohistochemistry , after sub-acute exposures of the zebrafish model,” *Environ. Toxicol. Pharmacol.*, vol. 34, no. 1, pp. 34–45, 2012.

[8] N. Laville, “Effects of human pharmaceuticals on cytotoxicity , EROD activity and ROS production in fish hepatocytes,” vol. 196, pp. 41–55, 2004.

[9] A. P. A. R. Robinson, J. A. B. B. Elden, and M. I. J. L. Ydy, “Toxicity of fluoroquinolone antibiotics to aquatic organisms,” vol. 24, no. 2, pp. 423–430, 2005.

[10] J. Kim, J. Park, C. Lee, K. Choi, and K. Choi, “Implication of global environmental changes on chemical toxicity- effect of water temperature , pH , and ultraviolet B irradiation on acute toxicity of several pharmaceuticals in Daphnia magna,” pp. 662–669, 2010.

[11] E. M. Smith *et al.*, “In vitro inhibition of cytochrome P450-mediated reactions by gemfibrozil , erythromycin , ciprofloxacin and fluoxetine in fish liver microsomes,” *Aquat. Toxicol.*, vol. 109, pp. 259–266, 2012.

[12] S. V Kergaravat, S. R. Hernandez, and A. M. Gagneten, “Second-, third- and fourth-generation quinolones: Ecotoxicity effects on Daphnia and Ceriodaphnia species,” *ECSN*, p. 127823, 2020.

[13] K. Eguchi, H. Nagase, M. Ozawa, K. Miyamoto, and H. Yoshimura, “Evaluation of antimicrobial agents for veterinary use in the ecotoxicity test using microalgae,” vol. 57, pp. 1733–1738, 2004.

[14] T. Ando, “A novel method using cyanobacteria for ecotoxicity test of veterinary antimicrobial agents,” vol. 26, no. 4, pp. 601–606, 2007.

[15] G. Lu, “Effects of selected pharmaceuticals on growth, reproduction and feeding of Daphnia Magna,” *Fresenius Environ. Bull.*, vol. 22(9), pp. 2583–2589, 2013.

[16] J. Liu, G. Lu, D. Wu, and Z. Yan, “Ecotoxicology and Environmental Safety A multi-biomarker assessment of single and combined effects of nor fl oxacin and sulfamethoxazole on male gold fish (*Carassius auratus*),” *Ecotoxicol. Environ. Saf.*, vol. 102, pp. 12–17, 2014.

[17] L. Yang, “Growth-inhibiting effects of 12 antibacterial agents and their mixtures on the freshwater microalga,” vol. 27, no. 5, pp. 1201–1208, 2008.

[18] J. Vestel, “Use of acute and chronic ecotoxicity data in environmental risk assessment of pharmaceuticals,” vol. 35, no. 5, pp. 1201–1212, 2016.

[19] N. Harada, K. Higuchi, and T. Yoshiyama, “Comparison of the sensitivity and specificity of two whole blood interferon-gamma assays for M . tuberculosis infection,” pp. 348–353, 2008.

[20] M. González-Pleiter *et al.*, “Toxicity of five antibiotics and their mixtures towards photosynthetic aquatic organisms: Implications for environmental risk assessment,” *Water Res.*, vol. 47, no. 6, pp. 2050–2064, 2013.

[21] M. R. de Orte, C. Carballeira, I. G. Viana, and A. Carballeira, “Assessing the toxicity of chemical compounds associated with marine land-based fish farms: The use of mini-scale microalgal toxicity tests,” *Chem. Ecol.*, vol. 29, no. 6, pp. 554–563, 2013.

[22] R. Oliveira, S. McDonough, J. C. L. Ladewig, A. M. V. M. Soares, A. J. A. Nogueira, and I. Domingues, “Effects of oxytetracycline and amoxicillin on development and biomarkers activities of zebrafish (Danio rerio),” *Environ. Toxicol. Pharmacol.*, vol. 36, no. 3, pp. 903–912, 2013.

[23] H. Qian *et al.*, “Analyses of gene expression and physiological changes in Microcystis aeruginosa reveal the phytotoxicities of three environmental pollutants,” *Ecotoxicology*, vol. 21, no. 3, pp. 847–859, 2012.

[24] K. O. Kusk, A. M. Christensen, and N. Nyholm, “Algal growth inhibition test results of 425 organic chemical substances,” *Chemosphere*, vol. 204, pp. 405–412, 2018.

[25] M. De Liguoro, V. Di Leva, M. Dalla Bona, R. Merlanti, G. Caporale, and G. Radaelli, “Sublethal effects of trimethoprim on four freshwater organisms,” *Ecotoxicol. Environ. Saf.*, vol. 82, pp. 114–121, 2012.
